# Establishment of an antepartum predictive model for postpartum hemorrhage in preterm delivery: a retrospective matched case-control study

**DOI:** 10.3389/fmed.2026.1841512

**Published:** 2026-08-20

**Authors:** Qiuping Liao, Baomei Xu, Lin Lin, Lianghui Zheng, Jinying Luo, Qian Yu, Jianying Yan

**Affiliations:** 1 College of Clinical Medicine for Obstetrics & Gynecology and Pediatrics, Fujian Medical University Fujian Maternity and Child Health Hospital, Fuzhou, Fujian, China; 2 Fujian Clinical Research Center for Maternal-Fetal Medicine, Fuzhou, Fujian, China; 3 Laboratory of Maternal-Fetal Medicine, Fujian Maternity and Child Health Hospital, Fuzhou, Fujian, China; 4 National Key Obstetric Clinical Specialty Construction Institution of China, Fuzhou, Fujian, China; 5 Department of Gynecology and Obstetrics, Xiang'an Hospital of Xiamen University, Xiamen, Fujian, China

**Keywords:** postpartum hemorrhage, predictive model, preterm delivery, risk factors, severe maternal morbidity

## Abstract

**Background:**

Postpartum hemorrhage (PPH) occupies a prominent position among severe maternal morbidity (SMM) in preterm pregnancies, yet current research on predicting PPH in preterm delivery remains limited.

**Aim:**

To establish an antepartum predictive model for postpartum hemorrhage in preterm delivery.

**Methods:**

This study included 728 singleton pregnant women. Clinical data of women were collected. Logistic regression analysis was performed to identify risk factors for PPH in preterm delivery, and a nomogram prediction model was established. Additionally, based on the basic principles and methods of the Back Propagation (BP) neural network and Garson algorithm, the importance ranking of variables was conducted.

**Results:**

Number of induced abortions≥2 [odds ratio (OR) = 2.067, 95%CI:1.192–3.585], gestational diabetes mellitus (GDM) (OR = 1.702, 95% CI: 1.111–2.607), placenta accreta (OR = 7.443, 95% CI: 3.955–14.005), placenta increta (OR = 60.170, 95% CI: 18.271–198.147), anemia (OR = 1.941, 95% CI: 1.303–2.891), and polyhydramnios (OR = 3.539, 95% CI: 1.202–10.417) were identified as risk factors for PPH in preterm delivery. The nomogram prediction model was constructed. The receiver operating characteristic (ROC) curve area under the curve (AUC) for the predictive model was 0.770. The Hosmer-Lemeshow test statistic χ^2^ = 1.708, *P* = 0.789. Following internal validation, the bias-corrected line approached the ideal line, with a consistency index (C-index) of 0.770. Using a BP neural network and Garson algorithm, the top three risk factors most significantly influencing PPH in preterm delivery were identified as placenta increta, placenta accreta, and polyhydramnios.

**Conclusion:**

This predictive model demonstrated moderate predictive ability in the derivation cohort, and its validation similarly showed acceptable performance. This model may be employed to identify women with high-risk factors of PPH in preterm delivery and to facilitate antepartum decision-making clinically.

## 1 Background

Preterm delivery is defined as delivery before 37 completed weeks of gestation, though its lower limit lacks a universally agreed definition. According to Chinese guidelines, the lower limit for preterm delivery is set at 28 completed weeks of gestation or a neonatal birth weight of at least 1,000 grams (1). World Health Organization (WHO) data show that the global preterm delivery rate was 9.9% in 2020 (2). In recent years, with adjustments to China's birth policies and widespread use of assisted reproductive technologies, the characteristics of the childbearing population and the spectrum of pregnancy-related diseases have been changing continuously. The proportion of pregnant women with advanced maternal age, twin or multiple pregnancies, hypertensive disorders of pregnancy (HDP), and gestational diabetes mellitus (GDM) has been increasing.

While the increase in neonatal morbidities associated with early preterm delivery have been well established (3), and many associations and modifiable behaviors and conditions have been identified for the care of the patient at risk for preterm delivery (4), studies evaluating the accompanying risks of maternal morbidities, particularly in the absence of maternal medical indications for preterm delivery, are limited. This was, in fact, highlighted in a joint workshop by the National Institute of Child Health and Development, Society for Maternal-Fetal Medicine, American Academy of Pediatrics, and American College of Obstetricians and Gynecologists, identifying short- and long-term maternal morbidities associated with preterm delivery as a key area for future research (5). Preterm delivery is associated with serious, potentially life-threatening complications during delivery, such as severe postpartum hemorrhage (s-PPH) and peripartum hysterectomy (6). Scholars have found, controlling for age, body mass index (BMI) greater than 30 kg/m^2^, nulliparity, insurance status, race, smoking status, and cesarean delivery, preterm deliveries were associated with significantly higher rates of postpartum hemorrhagic composite maternal morbidity [CMM, blood loss >1,000 mL, uterotonic use, transfusion, uterine tamponade, surgical intervention, intensive care unit (ICU) admission, hysterectomy, and maternal death] than those who delivered at term (7).

Kilpatrick et al. (8) found that preterm delivery is significantly associated with the incidence of severe maternal morbidity (SMM), with postpartum hemorrhage (PPH) being the most common cause, accounting for approximately 43.5%. Reddy et al. also reported an increased risk of SMM in early preterm delivery, particularly among women delivering by cesarean section (vaginal delivery vs. cesarean delivery: 23% vs. 3.5%). Some studies (9) have indicated that establishing a predictive early warning system for significant diseases, monitoring pregnant women with high-risk factors and quantifying and scoring these high-risk factors can provide early identification, timely intervention, and treatment and help reduce the occurrence of PPH. The nomogram (10) is a reliable statistical model that provides individualized and highly accurate risk assessment and is now widely used in obstetrics and gynecology. However, few studies have reported risk prediction models for PPH specifically in the preterm delivery population. In this study, the prediction of the risk of PPH in preterm delivery was conveniently and visually represented in the form of a nomogram. The objective of this study was to develop a more accurate, stable, and applicable model to predict the risk of PPH in preterm delivery before labor. We believe that our study can be used to strengthen prenatal management and evaluation as well as provide a reference for the prevention of the risk of PPH in preterm delivery and appropriate intervention strategies.

## 2 Methods

### 2.1 Population

This study adhered to the principles outlined in the Declaration of Helsinki and was approved by the Ethics Committee of Fujian Maternity and Child Health Hospital, Fuzhou, Fujian, China (2024KY204). A retrospective cohort study was conducted at the Department of Obstetrics at Fujian Maternity and Child Health Hospital, Fuzhou, China from January 2014 to December 2020. This was a retrospective matched case-control study. A total of 728 women with singleton preterm deliveries were selected as the study population, including the PPH group and the Non-PPH group. During the study period, 403 women experienced PPH after preterm delivery, after excluding those with multiple pregnancies (*n* = 105), gestational age < 28 weeks (*n* = 36), fetal anomalies or stillbirths (*n* = 30), incomplete clinical data (*n* = 11), and uterine rupture (*n* = 2), 219 women were ultimately included in the PPH group. For the Non-PPH group, eligible cases without PPH during the same period were individually matched by gestational age (within ± 6 days) and delivery date (within ± 1 months). Up to three non-PPH women were matched without replacement to one PPH woman; once a woman without PPH was selected, the same woman could not be used. A total of 509 women were finally enrolled in the Non-PPH group.

### 2.2 Measures

The diagnostic criteria for preterm delivery was defined as follows: delivery at a gestational age of 28 weeks or more, or neonatal birth weight of at least 1,000 g but less than 37 weeks gestation (1).

Postpartum hemorrhage (PPH) was defined according to the 25th edition of *Williams Obstetrics*. PPH is characterized by blood loss of 500 mL or more within 24 h following vaginal delivery, or 1,000 mL or more following cesarean section. Postpartum blood loss was routinely measured using a combination of the gravimetric method and volumetric method. Although the recent International Federation of Gynecology and Obstetrics (FIGO) recommendations adopt a uniform threshold of ≥1,000 mL for PPH. However, our country has consistently utilized the traditional definition (≥500 mL for vaginal delivery, ≥1,000 mL for cesarean section). We maintained this definition based on the need to maintain alignment with current national guidelines and local clinical practice. Furthermore, predicting a 500 mL blood loss provides significant clinical value as an early warning trigger, allowing clinicians to initiate prompt management.

Maternal data were extracted from the electronic health record system and included age, pre-pregnancy body mass index (BMI), gravidity, parity, gestational age at delivery, educational level, history of induced abortion, history of PPH, conception method, and pregnancy complications and comorbidities. These encompassed scarred uterus, HDP, GDM, placenta previa, placenta accreta spectrum disorders (PAS), intrahepatic cholestasis of pregnancy (ICP), thyroid dysfunction, hepatitis B surface antigen carrier status, anemia, polyhydramnios, premature rupture of membranes (PROM), and administration of magnesium sulfate within 24 h prior to delivery. It should be emphasized that PAS was defined as the presence of placenta previa with placental invasion identified by prenatal ultrasound or magnetic resonance imaging (MRI), accompanied by at least one of the following clinical manifestations at or after delivery: (1) Partial or complete failure of manual placental separation, with absence of a cleavage plane between part or all of the placenta and the uterine wall; (2) Severe hemorrhage at the implantation site following forced manual placental separation, without alternative etiologies for PPH; (3) Histopathological confirmation of PAS in hysterectomy specimens; (4) Typical intraoperative findings of PAS detected during delivery exploration in patients with suspected PAS on prenatal imaging. Placenta accreta refers to villous invasion into the superficial uterine myometrium, whereas placenta increta is defined as villous invasion extending into the deep uterine myometrium.

### 2.3 Statistical analysis

Data processing, analysis, and visualization were performed using SPSS version 26.0 and R version 4.2.2. Measurement data with a normal distribution were presented as mean ± standard deviation ($\bar{x}\pm s$), while non-normally distributed measurement data were described using median and interquartile range (IQR). Homogeneity of variance was tested for measurement data; if variances were equal, analysis of variance (ANOVA) was applied, otherwise nonparametric tests were used. Group comparisons for measurement variables were conducted using either the *t*-test or Mann-Whitney U test, according to their distribution characteristics. Categorical data were expressed as frequency (*n*) and percentage (%), and compared using the chi-square test or Fisher's exact test.

Variables with *P* < 0.1 in univariate analysis were included in multivariate logistic regression for further investigation. Forward stepwise logistic regression was employed to identify risk factors for PPH in preterm delivery, with calculation of *P* values and 95% confidence intervals (CI) for odds ratios (OR). All statistical tests were two-sided, with *P* < 0.05 considered statistically significant. Prior to conducting the multivariate logistic regression, multicollinearity among the selected variables was assessed using the value of Variance Inflation Factor (VIF). A VIF value of less than 5 was considered indicative of no significant multicollinearity. For the later variable selection of the prediction model, most predictors were screened based on statistically significant factors from multivariate regression analysis, while one variable with *P* > 0.05 was retained based on clinical evidence and published literatures.

Subsequently, based on the fundamental principles and methods of the backpropagation (BP) neural network and Garson's algorithm, a BP neural network was established to rank variable importance. The model topology is 7-10-1: the input layer contains 7 neurons (corresponding to 7 risk factors), the hidden layer contains 10 neurons, and the output layer contains 1 neuron. Bias neurons are included in both the input and hidden layers. The model training parameters are set as follows: both the hidden layer and the output layer use the logistic (sigmoid) activation function; the rprop+ algorithm is used for training, with the error threshold set to 0.05 and a maximum of 100,000 training iterations. The model is independently trained 10 times with different initializations, and the model with the lowest training error is selected as the final model. To prevent overfitting, two control measures are adopted: (i) Early stopping: training is automatically halted when the training error falls below the preset threshold of 0.05; (ii) 5-fold cross-validation: 5-fold cross-validation is performed on the entire training set, and the average performance is taken as the final model performance. During the experiment, the random seed is fixed at 812 to ensure reproducibility.

The multivariate logistic regression model was visualized using the *rms* package in R 4.2.2 to develop a nomogram prediction model. The model's discrimination, calibration, and clinical utility were evaluated by plotting the receiver operating characteristic (ROC) curve, calibration curve, and clinical decision curve, respectively. Internal validation was performed using bootstrap resampling with 1,000 iterations.

This development study of a clinical predictive nomogram was reported following the Transparent Reporting of a multivariable prediction model for Individual Prognosis Or Diagnosis (TRIPOD) reporting guideline. Given only internal bootstrap validation was conducted in our work, relevant items for external validation in TRIPOD were not applicable.

## 3 Results

### 3.1 Baseline characteristics of all participants

Table 1 shows that the differences in age, pre-pregnancy BMI, gravidity, parity, and educational level between the two groups were statistically significant (*P* < 0.05), while the difference in gestational age at delivery was not statistically significant (*P* > 0.05).

**Table 1:** Comparison of clinical characteristics.

Characteristics	PPH group (*N* = 219) Mean (SD)/medianor *n* (%)	Non-PPH group (*N* = 509) Mean (SD)/medianor *n* (%)	*P-*value
Maternal age (year)	31.68 ± 4.91	29.91 ± 4.84	< 0.001^*^
Pre-pregnancy BMI (kg/m^2^)	21.67 ± 3.13	21.00 ± 4.02	0.036^*^
Gravidity	3.10 ± 1.57	2.31 ± 1.43	< 0.001^*^
Parity	1.79 ± 0.70	1.54 ± 0.65	< 0.001^*^
Gestational age at delivery (weeks)	34.15 ± 2.12	34.11 ± 2.15	0.823
Education level			0.015^*^
Junior school education or less	62 (28.3)	96 (18.9)	
Senior school education	74 (33.8)	183 (36.0)	
University degree or above	83 (37.9)	230 (45.2)	

Continuous variables conforming to normal distribution are presented as mean ± standard deviation and compared using independent-samples Student's *t*-test; non-normally distributed continuous data are expressed as median (interquartile range) and analyzed with Mann-Whitney U test. Categorical variables are shown as *n* (%) and compared by χ^2^ test or Fisher's exact test when appropriate.

BMI, body mass index. Statistically significant: ^*^P < 0.05.

### 3.2 Risk factors of postpartum hemorrhage in preterm delivery

All factors were individually subjected to univariate analysis. The results showed that advanced maternal age, pre-pregnancy overweight, educational level, multiparity, history of induced abortion, previous PPH, scarred uterus, placenta previa, PAS, anemia, and polyhydramnios were significantly associated with PPH in preterm delivery (*P* < 0.05). Conversely, conception method, HDP, GDM, ICP, thyroid dysfunction, hepatitis B surface antigen carrier status, PROM, and history of magnesium sulfate use were not significantly associated (*P* > 0.05), as shown in Table 2.

**Table 2:** Univariate analysis to explore the risk factors.

Variables	PPH group (*N* = 219) *n* (%)	Non-PPH group (*N* = 509) *n* (%)	*χ^2^*	*P*-Value
Maternal age (year)			13.904	< 0.001^*^
< 35	158 (72.1)	428 (84.1)		
≥35	61 (27.9)	81 (15.9)		
Overweigh (BMI ≥ 24 kg/m^2^)			4.351	0.037^*^
No	171 (78.1)	430 (84.5)		
Yes	48 (21.9)	79 (15.5)		
Education level			8.394	0.015^*^
Junior school education or less	62 (28.3)	96 (18.9)		
Senior school education	74 (33.8)	183 (36.0)		
University degree or above	83 (37.9)	230 (45.2)		
Parity			17.936	< 0.001^*^
0	80 (36.5)	273 (53.6)		
≥1	139 (63.5)	236 (46.4)		
Number of induced abortions			34.961	< 0.001^*^
0	117 (53.4)	365 (71.7)		
1	50 (22.8)	100 (19.6)		
≥2	52 (23.7)	44 (8.6)		
History of PPH			14.983	< 0.001^*^
No	211 (96.3)	508 (99.8)		
Yes	8 (3.7)	1 (0.2)		
Conception method			0.876	0.349
Natural conception	207 (94.5)	489 (96.1)		
Assisted reproduction for pregnancy	12 (5.5)	20 (3.9)		
Scar uterus			30.997	< 0.001^*^
No	133 (60.7)	409 (80.4)		
Yes	86 (39.3)	100 (19.6)		
HDP			0.450	0.502
No	187 (85.4)	444 (87.2)		
Yes	32 (14.6)	65 (12.8)		
GDM			3.048	0.081
No	160 (73.1)	402 (79)		
Yes	59 (26.9)	107 (21)		
Placenta previa			112.279	< 0.001^*^
No	138 (63.0)	478 (93.9)		
Yes	81 (37.0)	31 (6.1)		
Placental accretic diseases			183.270	< 0.001^*^
No	126 (57.5)	490 (96.3)		
Placenta accreta	41 (18.7)	16 (3.1)		
Placenta increta	52 (23.7)	3 (0.6)		
ICP			0.010	0.921
No	212 (96.8)	492 (96.7)		
Yes	7 (3.2)	17 (3.3)		
Thyroid dysfunction			3.349	0.163
No	212 (96.8)	502 (98.6)		
Hypothyroidism	5 (2.3)	6 (1.2)		
Hyperthyroidism	2 (0.9)	1 (0.2)		
HBsAg Carrier			0.233	0.629
No	457 (89.8)	52 (10.2)		
Yes	194 (88.6)	25 (11.4)		
Anemia			26.983	< 0.001^*^
No	125 (57.1)	388 (76.2)		
Yes	94 (42.9)	121 (23.8)		
Polyhydramnios			6.836	0.009^*^
No	209 (95.4)	502 (98.6)		
Yes	10 (4.6)	7 (1.4)		
PROM			2.534	0.111
No	134 (61.2)	279 (54.8)		
Yes	85 (38.8)	230 (45.2)		
Magnesium sulfate use			0.076	0.783
No	149 (68.0)	341 (67.0)		
Yes	70 (32.0)	168 (33.0)		

Categorical variables were compared using the χ^2^ test; Fisher's exact test was adopted when theoretical frequency was insufficient.

BMI, Body mass index; PPH, Postpartumhemorrhage; HDP, Hypertensive disorders of pregnancy; GDM, Gestational diabetes mellitus; HbsAg, Hepatitis B surface antigen; ICP, Intrahepatic cholestasis of pregnancy; PROM, Premature rupture of membranes. Statistically significant: ^*^P < 0.05.

Variables with *P* < 0.1 in the univariate analysis were included in the multivariate logistic regression analysis. The results indicated that a history of two or more induced abortions (OR = 2.067, 95% CI: 1.192–3.585), GDM (OR = 1.702, 95% CI: 1.111–2.607), placenta accreta (OR = 7.443, 95% CI: 3.955–14.005), placenta increta (OR = 60.170, 95% CI: 18.271–198.147), anemia (OR = 1.941, 95% CI: 1.303–2.891), and polyhydramnios (OR = 3.539, 95% CI: 1.202–10.417) were independent risk factors for PPH in preterm delivery. These results are presented in Table 3. Although the variable “one induced abortion” presented *P* > 0.05 in multivariate analysis, clinical observations and existing studies have confirmed its association with PPH, as presented in the Discussion. Therefore, this variable was incorporated into both the final nomogram and BP neural network.

**Table 3:** Multivariate logistic regression analysis to explore the independent risk factors.

Variables	PPH group (*N* = 219) *n* (%)	Non-PPH group (*N* = 509) *n* (%)	OR	95% CI	*P*-value	VIF
Number of induced abortions						1.068
0	117 (53.4)	365 (71.7)		Ref		
1	50 (22.8)	100 (19.6)	1.239	0.778–1.972	0.367	
≥2	52 (23.7)	44 (8.6)	2.067	1.192–3.585	0.010^*^	
GDM						1.008
No	160 (73.1)	402 (79)		Ref		
Yes	59 (26.9)	107 (21)	1.702	1.111–2.607	0.015^*^	
PAS						1.091
No	126 (57.5)	490 (96.3)		Ref		
Placenta accreta	41 (18.7)	16 (3.1)	7.443	3.955–14.005	< 0.001^*^	
Placenta increta	52 (23.7)	3 (0.6)	60.170	18.271–198.147	< 0.001^*^	
Anemia						1.041
No	125 (57.1)	388 (76.2)		Ref		
Yes	94 (42.9)	121 (23.8)	1.941	1.303–2.891	< 0.001^*^	
Polyhydramnios						1.006
No	209 (95.4)	502 (98.6)		Ref		
Yes	10 (4.6)	7 (1.4)	3.539	1.202–10.417	0.022^*^	

Binary logistic regression analysis was used to explore risk factors. Dependent variable: postpartum hemorrhage (0 = no, 1 = yes); all independent variables were categorical variables, and odds ratio (OR) with 95% confidence interval (95% CI) and *P* values were calculated. Multicollinearity among the selected variables was assessed using the value of VIF.

GDM, Gestational diabetes mellitus; PAS, Placenta accreta spectrum disorders; VIF, Variance Inflation Factor.

Statistically significant: ^*^P < 0.05.

**Figure 1:**
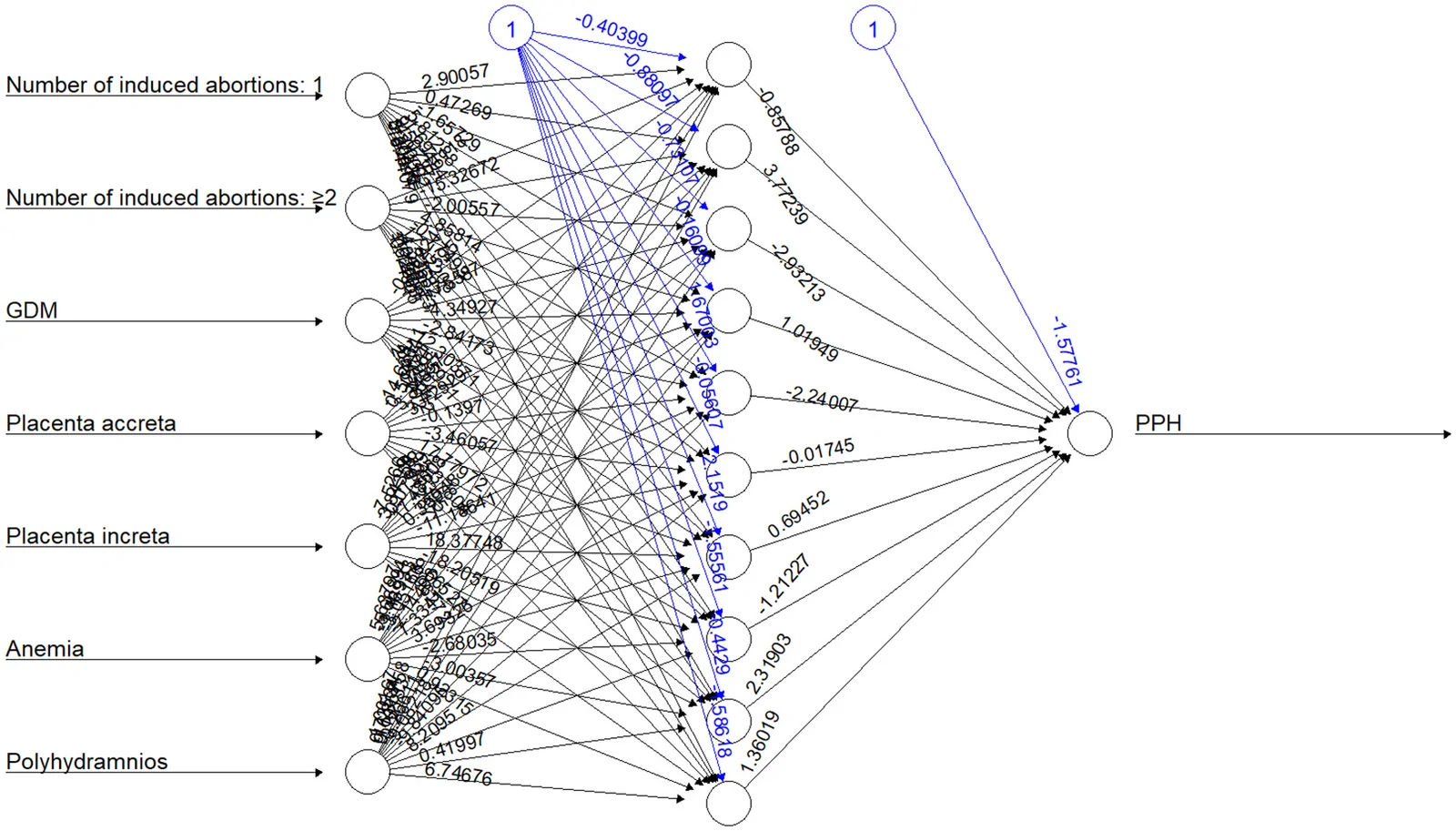
Structure diagram of BP neural network. The neural network was constructed with the select variables as inputs. BP, Back Propagation; PPH, postpartum hemorrhage.

**Figure 2:**
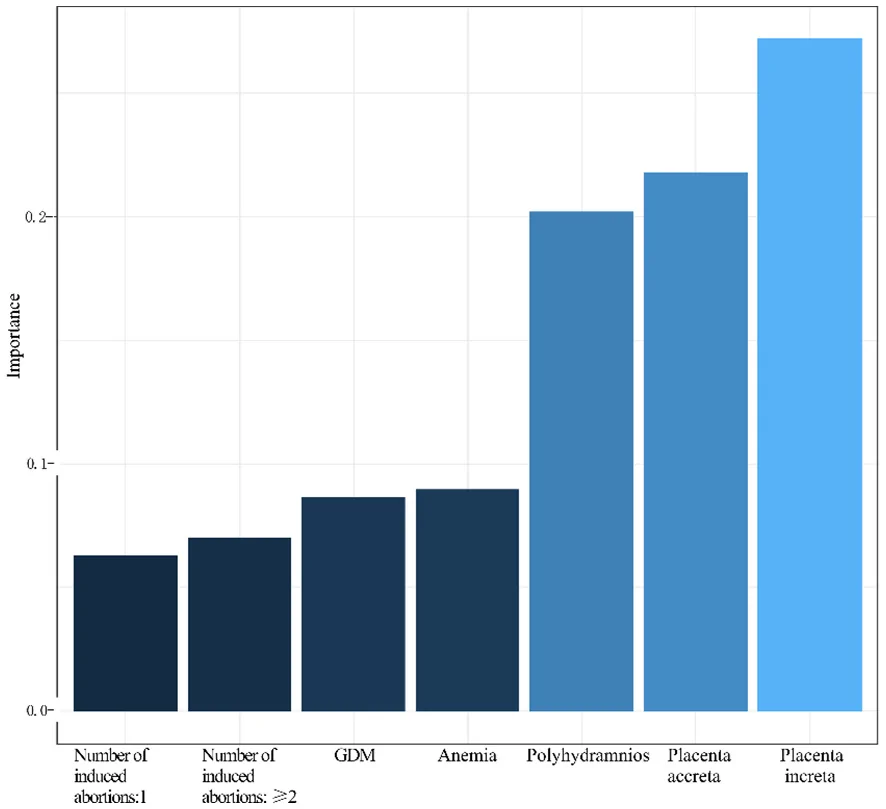
Ranking the importance of risk factors for PPH in preterm delivery. The relative importance of each input variable was calculated using Garson's algorithm based on the established BP neural network. The top three most influential predictors identified were placenta increta, placenta accreta, and polyhydramnios, which is consistent with the findings from the multivariate logistic regression analysis. PPH, postpartum hemorrhage.

Based on Garson's algorithm, the importance of the select variables was ranked (Figure 1). Using Garson's algorithm to analyze the network, the top three most influential factors identified were placenta increta, placenta accreta, and polyhydramnios (Figure 2).

### 3.3 Construction of the nomogram prediction model

**Figure 3:**
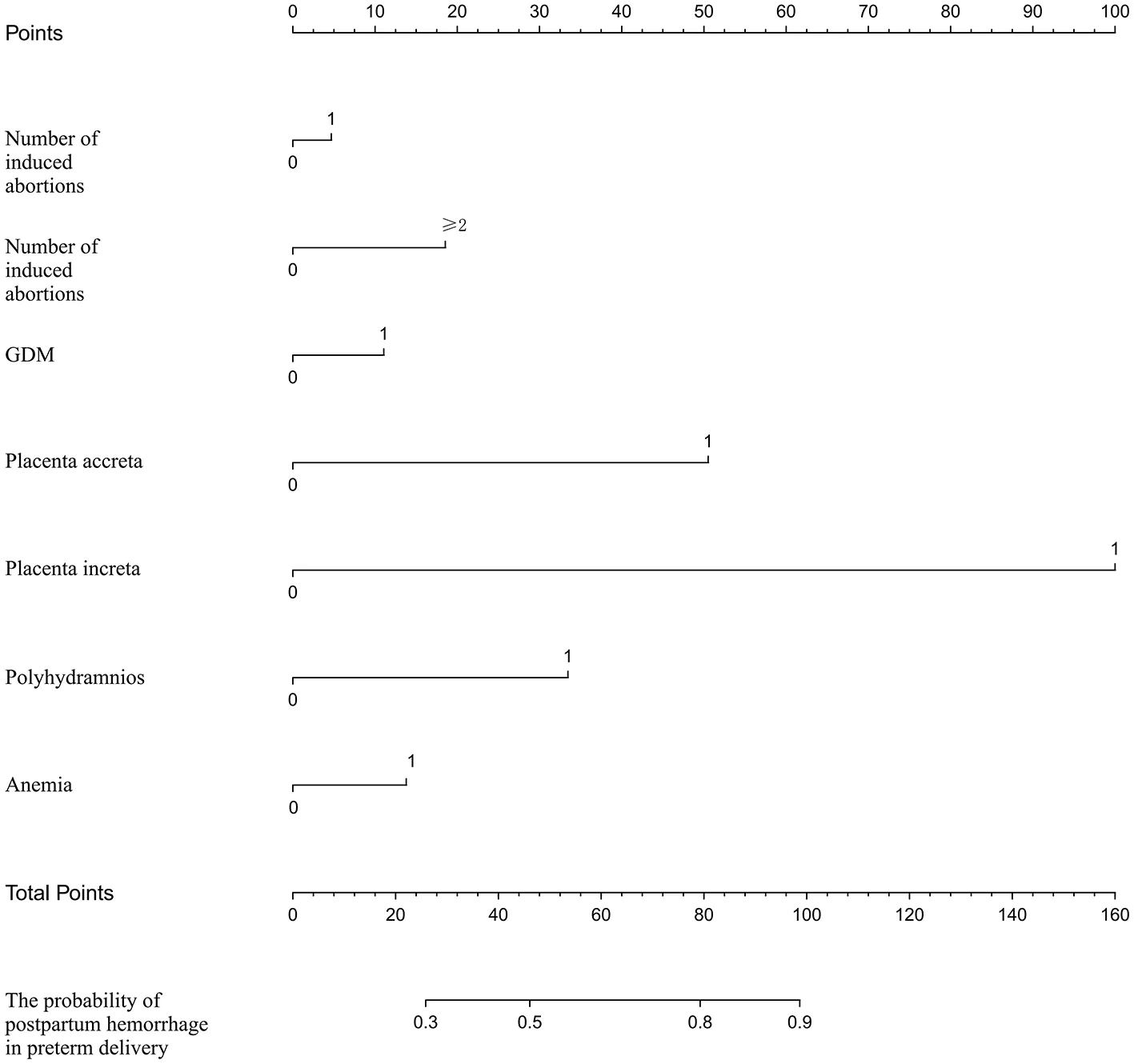
Nomogram for predicting the risk of PPH in preterm delivery. The nomogram was constructed based on the select variables. To use the nomogram, locate the patient's value on each variable axis, draw a vertical line upwards to the “Points” axis to get a score, and sum all scores on the “Total Points” axis to determine the individual predicted probability of PPH. The Hosmer-Lemeshow test indicated a good model fit (χ^2^ = 1.708, *P* = 0.789). PPH, postpartum hemorrhage; GDM, gestational diabetes mellitus.

A nomogram risk prediction model was constructed (Figure 3). The Hosmer-Lemeshow test statistic was χ^2^ = 1.708 with *P* = 0.789, indicating a good fit of the model to the data.

**Figure 4:**
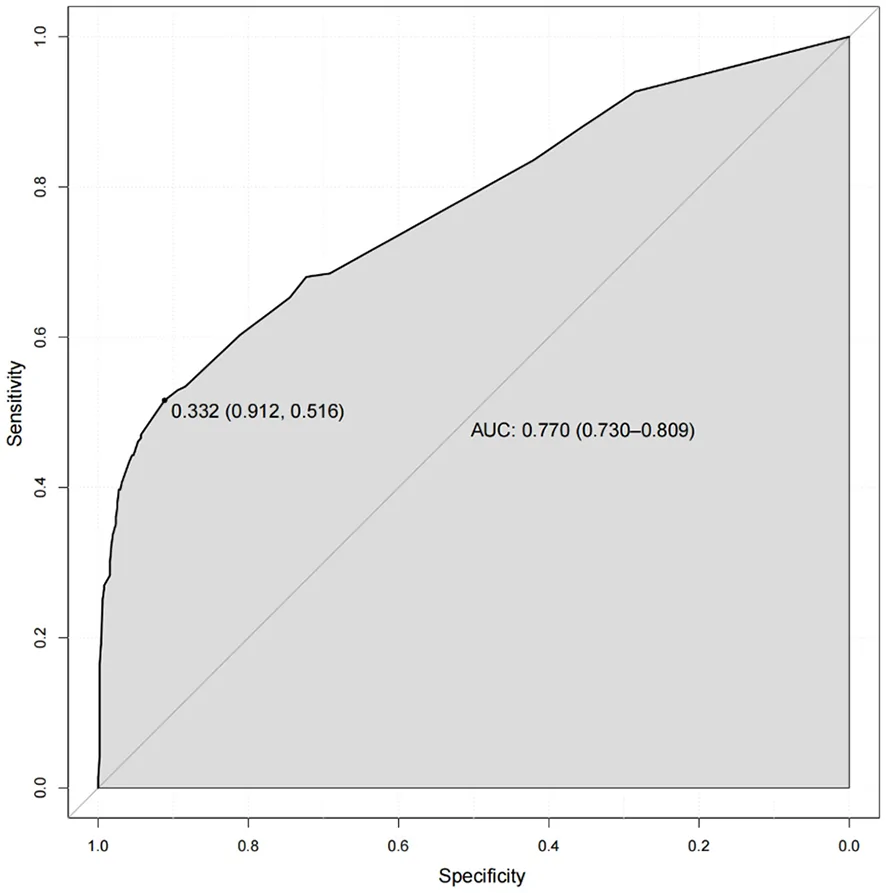
ROC curve evaluating the discriminative ability of the nomogram prediction model. The area under the curve (AUC) is 0.770 (95% CI: 0.730–0.809). At the optimal cutoff value of 0.332, the Youden index is 0.428, suggesting that the model possesses good discriminative capability for predicting PPH. ROC, receiver operating characteristic; AUC, area under the curve; CI, confidence interval; PPH, postpartum hemorrhage.

The ROC curve of the prediction model was plotted, yielding an area under the curve (AUC) of 0.770 (95% CI: 0.730–0.809) and a Youden index of 0.428 at a cutoff value of 0.332, suggesting moderate discriminative ability of the model (Figure 4).

**Figure 5:**
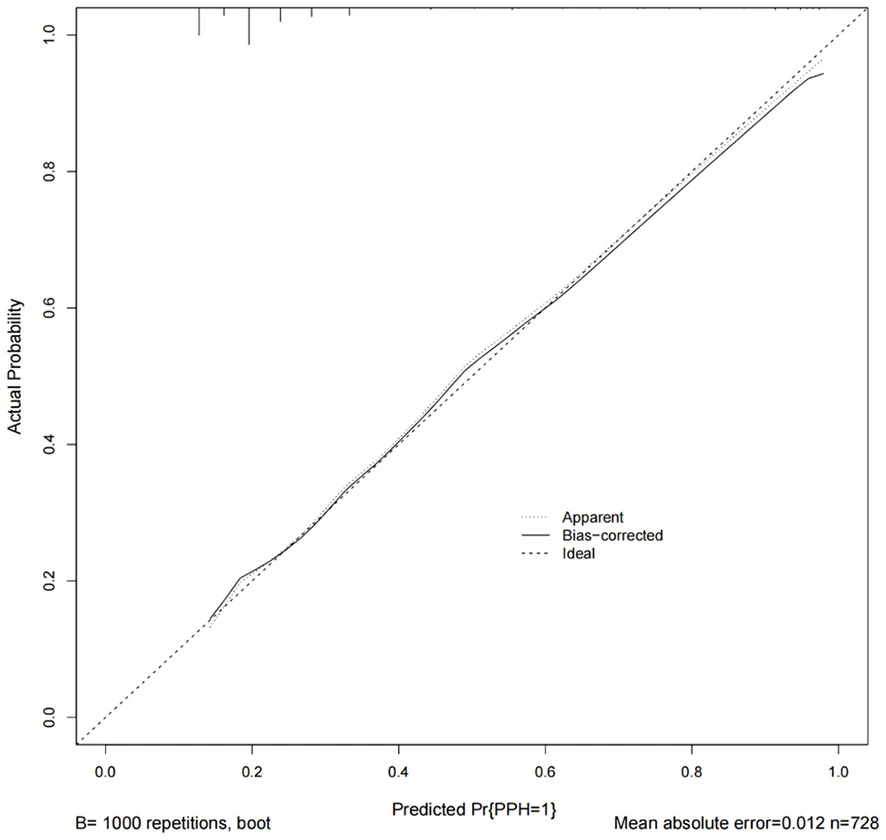
Calibration curve of the prediction model for PPH in preterm delivery. Internal validation was performed using bootstrap resampling with 1,000 iterations. The x-axis represents the nomogram-predicted probability of PPH, and the y-axis represents the actual observed rate. The diagonal solid line represents the performance of an ideal model. The bias-corrected curve closely approximates the ideal line with a concordance index (C-index) of 0.770 and a mean absolute error of 0.012, indicating excellent calibration of the model. PPH, postpartum hemorrhage.

Internal validation using bootstrap resampling with 1,000 iterations showed that the bias-corrected calibration curve closely approximated the ideal line, with a concordance index (C-index) of 0.770. The calibration plot demonstrated a mean absolute error of 0.012 between predicted and observed values, indicating good calibration of the model (Figure 5).

**Figure 6:**
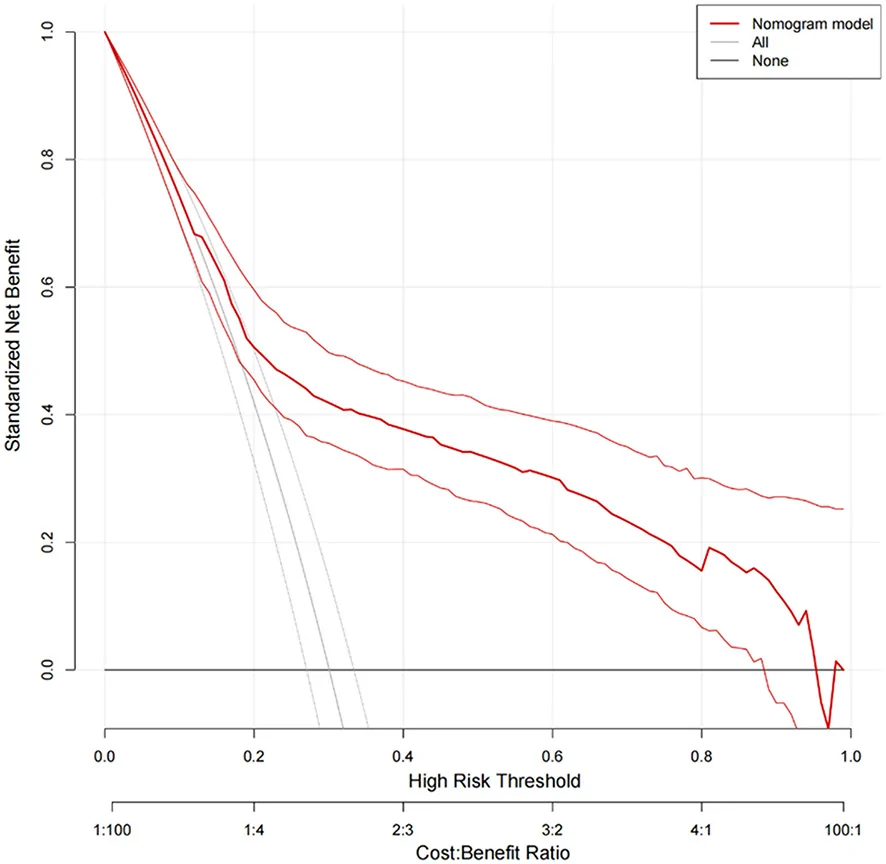
Decision curve analysis (DCA) assessing the clinical utility of the prediction model. The x-axis indicates the threshold probability, and the y-axis indicates the net benefit. The decision curve demonstrates that the net benefit of the prediction model remains above both extreme lines (the “treat all” and “treat none” assumptions) across a wide threshold probability range from 0.13 to 0.95, indicating that the use of this nomogram is clinically beneficial for antepartum decision-making. DCA, decision curve analysis.

In the decision curve analysis, the net benefit curve was above both extreme lines across a threshold probability range of 0.13 to 0.95, indicating that the model has clinical utility (Figure 6).

## 4 Discussion

In this study, a retrospective analysis was conducted on the clinical data of 728 women with singleton preterm deliveries. Multivariate analysis identified a history of induced abortion (≥2), GDM, PAS, anemia, and polyhydramnios as independent risk factors for PPH in preterm deliveries. Based on these predictors, an antepartum nomogram was successfully constructed and validated. The model demonstrated moderate discrimination and calibration, with an area under the curve (AUC) of 0.770 and a Hosmer-Lemeshow test *P*-value of 0.789. Internal validation further confirmed the robustness of the model, yielding a concordance index (C-index) of 0.770. Decision curve analysis (DCA) indicated that the nomogram possesses clinical utility within a threshold probability range of 0.13 to 0.95. Furthermore, this study innovatively utilized a BP neural network combined with Garson's algorithm to quantify the relative importance of each risk factor. The results revealed that placenta increta, placenta accreta, and polyhydramnios were the top three contributors to the model.

In the present study, PAS were identified as the primary determinants of PPH in preterm deliveries. Notably, placenta increta (OR = 60.170) and placenta accreta (OR = 7.443) exhibited exceptionally high odds ratios and ranked highest in importance according to the neural network weight analysis. These findings align closely with previous literature (11). In cases of preterm delivery, placental pathology often serves as a direct precipitating factor for the delivery itself. When placenta accreta or increta occurs, placental villi invade the myometrium or even penetrate the serosa. This invasion prevents spontaneous placental detachment after delivery and impairs myometrial contraction, thereby triggering catastrophic hemorrhage (12). Furthermore, since the lower uterine segment is often underdeveloped in preterm pregnancies, the contractility of muscle fibers is relatively weak. Consequently, achieving hemostasis becomes significantly more challenging when complicated by morbidly adherent placenta. A large retrospective cohort study by Matsuzaki et al. (13) demonstrated that the risks of PPH, disseminated intravascular coagulation (DIC), hemorrhagic shock, and maternal mortality escalate with the depth of placental villous invasion into the myometrium.

Polyhydramnios emerged as another significant risk factor in our study, ranking as the third most influential predictor in the BP neural network analysis. In a prospective cohort study, Vanda et al. (14) observed that the incidence of cesarean section, PPH, and neonatal asphyxia was significantly higher in the idiopathic polyhydramnios group compared to the normal pregnancy group. Furthermore, these risks were found to escalate in correlation with the severity of polyhydramnios. Polyhydramnios has been confirmed as an independent predictor of PPH; physiologically, it causes excessive stretching of uterine muscle fibers and increases uterine wall tension. This mechanical stress can induce myometrial degeneration or fiber rupture, ultimately resulting in uterine atony (15, 16). It is worth noting that despite the relatively smaller fetal dimensions of preterm fetuses, the increased intrauterine pressure caused by polyhydramnios often exerts a more profound impact than fetal weight itself. This implies that clinicians must remain vigilant regarding atonic hemorrhage caused by uterine overdistension in preterm pregnancies complicated by polyhydramnios, regardless of fetal dimensions.

Our study further demonstrated that a history of ≥2 induced abortions significantly increases the risk of PPH in preterm deliveries. In a prospective cohort study involving 3,026 pregnant women, Feleked et al. (17) reported that 23.1% of women with a history of abortion experienced antepartum hemorrhage, while 25.6% experienced PPH. Repeated intrauterine procedures can induce inflammatory responses and fibrotic repair in the endometrium and superficial myometrium. A 2020 study utilizing Shear Wave Elastography (SWE) confirmed a positive correlation between the number of induced abortions and endometrial stiffness. Increased endometrial stiffness indicates more severe scarring, which not only affects the placental implantation site (predisposing to placenta previa) but may also limit the physiological retraction of uterine muscle fibers after delivery, thereby leading to PPH caused by uterine atony (18). Furthermore, due to endometrial damage in the uterine fundus or corpus, the blastocyst may “migrate” to the lower uterine segment or cervical os in search of a suitable implantation environment, resulting in placenta previa. International cohort studies have shown a significant positive correlation between a history of non-medically indicated induced abortion and placenta-related complications in subsequent pregnancies, including placenta previa and placental abruption (aOR = 2.14) (19). Both conditions are established high-risk factors for PPH. Given the increasing application of assisted reproductive technology (ART) and the rising proportion of women of advanced maternal age, this risk factor warrants specific attention in the management of the preterm population.

Regarding the impact of GDM, although GDM did not exhibit statistical significance in the univariate analysis, it emerged as an independent risk factor in the multivariate model after adjusting for confounders. Inflammation and oxidative stress are implicated in the pathophysiology of GDM. Insulin resistance among pregnant women with GDM not only disturbs glucose and lipid metabolism but also alters the inflammatory microenvironment at the maternal-fetal interface, thereby elevating the risk of preterm delivery (20). GDM markedly increases the incidence of adverse perinatal outcomes and has been validated as an independent predictive factor for postpartum hemorrhage (PPH) (21). Persistent hyperglycemia contributes to PPH via multiple pathways: hyperglycemia-induced vascular endothelial injury impairs myometrial contractility; osmotic diuresis secondary to hyperglycemia leads to polyhydramnios and subsequent uterine atony; abnormal glucose metabolism disrupts the coagulation-fibrinolysis system; and excessive fetal growth aggravates birth-related trauma (22, 23).

Our study identified anemia as an independent risk factor for PPH in preterm delivery. Anemia (by causing hypoxia) and iron deficiency (by increasing serum norepinephrine concentrations) can induce maternal and fetal stress, which stimulates the synthesis of corticotropin-releasing hormone (CRH). Elevated CRH concentrations are a major risk factor for preterm labor, pregnancy-induced hypertension and eclampsia, and premature rupture of the membranes. An alternative mechanism could be that iron deficiency increases oxidative damage to erythrocytes and the fetoplacental unit. Iron deficiency may also increase the risk of maternal infections, which can stimulate the production of CRH and are a major risk factor for preterm delivery (24). The same hypoxic insult compromises uterine contractility during the third stage of labor. Inadequate oxygen delivery to the myometrium impairs mitochondrial oxidative phosphorylation in uterine smooth muscle cells, resulting in insufficient ATP production to sustain effective and sustained myometrial contraction after placental delivery (25). Blood flow from bleeding vessels is increased by a reduction in blood viscosity seen in association with a reduced hematocrit, combined with maternal tachycardia and increased cardiac output, both compensatory to maintaining oxygen delivery in anemia. Anemic blood clots are fragile and more susceptible to fibrinolysis, and chronic hypoxia can lead to myometrial ischemia and hypoxia or affect nitric oxide (NO) synthesis, resulting in uterine atony. This predisposes the mother to a vicious cycle of ‘hemorrhage-coagulopathy', which is the most common cause of PPH (26, 27).

Unlike the majority of previous studies that focused primarily on PPH prediction in term deliveries, the present study focuses exclusively on the preterm population. Although preterm delivery is inherently a high-risk factor for PPH, the hemorrhagic risk associated with non-medically indicated or late preterm deliveries is frequently underestimated in clinical practice due to the paucity of targeted assessment tools. The nomogram constructed herein translates complex statistical data into an intuitive visual clinical tool, enabling physicians to rapidly calculate individualized risk probabilities. ROC analysis demonstrated an AUC of 0.770, and DCA confirmed that the model yields a net clinical benefit across a wide range of threshold probabilities. As such, this tool may serve as a supplementary aid for guiding the antenatal preparation of blood products and the administration of prophylactic uterotonics.

Several limitations of this study must be acknowledged. First, due to the retrospective nature of the study design, the analysis is inherently susceptible to potential information bias. Second, given that the recent FIGO diagnostic criteria for PPH differ from those used in this study, the generalizability of our model requires further validation. However, adopting the recent FIGO threshold (≥1,000 mL regardless of delivery mode) would substantially reduce the number of positive cases, leading to a decrease in the model's sensitivity and an increase in its specificity. Furthermore, the model's predictive focus would shift from “early warning” to “severe hemorrhage prediction,” which might also change the relative importance of certain predictors. Further research will aim to evaluate and compare models based on different diagnostic criteria. Finally, as the data were derived from a single center, future multicenter, large-scale prospective cohort studies are warranted to externally validate the model and evaluate its generalizability across different populations.

## 5 Conclusion

This study revealed that a history of induced abortions, GDM, PAS disorders, anemia, and polyhydramnios were identified as critical risk factors for PPH in preterm deliveries. The prediction model developed in this study demonstrated moderate discrimination and calibration. It serves as an effective auxiliary tool for clinicians to identify high-risk individuals at an early stage, thereby facilitating the optimization of prenatal management strategies and improving maternal and neonatal outcomes.

## Data Availability

The raw data supporting the conclusions of this article will be made available by the authors, without undue reservation.

## References

[1] Subgroup Subgroup of Obstetrics Society Society of Obstetrics and Gynecology Chinese Medical Association. [Clinical guidelines for the prevention and treatment of preterm birth (version 2024)]. Zhonghua Fu Chan Ke Za Zhi. (2024) 59:257–69. doi: 10.3760/cma.j.cn112141-20231119-00208 38644272

[2] Ohuma EO Moller AB Bradley E Chakwera S Hussain-Alkhateeb L Lewin A . National, regional, and global estimates of preterm birth in 2020, with trends from 2010: a systematic analysis. Lancet. (2023) 402:1261–71. doi: 10.1016/S0140-6736(23)00878-4 37805217

[3] Manuck TA Rice MM Bailit JL Grobman WA Reddy UM Wapner RJ . Preterm neonatal morbidity and mortality by gestational age: a contemporary cohort. Am J Obstet Gynecol. (2016) 215:103.e1–14. doi: 10.1016/j.ajog.2016.01.004 PMC4921282 26772790

[4] Harris S Greene A Downs S Sakowicz A Quinn KH Denney JM. Preterm birth: thoughtful strategies for screening and management of risk factors: a descriptive review. Clin Exp Obstet Gynecol. (2024) 51. doi: 10.31083/j.ceog5105110

[5] Raju TN Mercer BM Burchfield DJ Joseph GF Jr. Periviable birth: executive summary of a joint workshop by the Eunice Kennedy Shriver National Institute of Child Health and Human Development, Society for Maternal-Fetal Medicine, American Academy of Pediatrics, and American College of Obstetricians and Gynecologists. Am J Obstet Gynecol. (2014) 210:406–17. doi: 10.1016/j.ajog.2014.02.027 24725732

[6] Gulersen M Lenchner E Goyal A Grunebaum A Chervenak FA Bornstein E. Maternal morbidity associated with early preterm birth in low-risk singleton pregnancies. J Clin Med. (2024) 13:7061. doi: 10.3390/jcm13237061 39685519 PMC11642920

[7] Whelan A Ciomperlik H Ghose I Mendez-Figueroa H Wagner S Wiley R. Postpartum hemorrhage: preterm versus term pregnancies [ID: 1356883]. Obstet Gynecol. (2023) 141:102S−3S. doi: 10.1097/01.AOG.0000931264.93401.76

[8] Kilpatrick SJ Abreo A Gould J Greene N Main EK. Confirmed severe maternal morbidity is associated with high rate of preterm delivery. Am J Obstet Gynecol. (2016) 215:233.e1–7. doi: 10.1016/j.ajog.2016.02.026 26899903

[9] Krishnamoorthy S Liu Y Liu K. A novel oppositional binary crow search algorithm with optimal machine learning based postpartum hemorrhage prediction model. BMC Pregnancy Childbirth. (2022) 22:560. doi: 10.1186/s12884-022-04775-z 35831804 PMC9281026

[10] Park SY. Nomogram: an analogue tool to deliver digital knowledge. J Thorac Cardiovasc Surg. (2018) 155:1793. doi: 10.1016/j.jtcvs.2017.12.107 29370910

[11] Escobar MF Nassar AH Theron G Barnea ER Nicholson W Ramasauskaite D . FIGO recommendations on the management of postpartum hemorrhage 2022. Int J Gynaecol Obstet. (2022) 157:3–50. doi: 10.1002/ijgo.14116 35297039 PMC9313855

[12] Bartels HC Postle JD Downey P Brennan DJ. Placenta accreta spectrum: a review of pathology, molecular biology, and biomarkers. Dis Markers. (2018) 2018:1507674. doi: 10.1155/2018/1507674 30057649 PMC6051104

[13] Matsuzaki S Mandelbaum RS Sangara RN McCarthy LE Vestal NL Klar M . Trends, characteristics, and outcomes of placenta accreta spectrum: a national study in the United States. Am J Obstet Gynecol. (2021) 225:534.e1–38. doi: 10.1016/j.ajog.2021.04.233 33894149

[14] Vanda R Bazrafkan M Rouhani M Bazarganipour F. Comparing pregnancy, childbirth, and neonatal outcomes in women with idiopathic polyhydramnios: a prospective cohort study. BMC Pregnancy Childbirth. (2022) 22:399. doi: 10.1186/s12884-022-04625-y 35546395 PMC9097041

[15] Balki M Wong CA. Refractory uterine atony: still a problem after all these years. Int J Obstet Anesth. (2021) 48:103207. doi: 10.1016/j.ijoa.2021.103207 34391025

[16] Society for Maternal-Fetal Medicine (SMFM) Dashe JS Pressman EK Hibbard JU. SMFM consult series #46: evaluation and management of polyhydramnios. Am J Obstet Gynecol. (2018) 219:B2–8. doi: 10.1016/j.ajog.2018.07.016 30048635

[17] Feleke BE Feleke TE Nigussie AA Misgan E. The effects of stillbirth and abortion on the next pregnancy: a longitudinal study. BMC Womens Health. (2021) 21:340. doi: 10.1186/s12905-021-01485-0 34563190 PMC8464111

[18] Jiao Y Xue N Zou C Shui X Wang H Hu C. Assessment of early damage of endometrium after artificial abortion by shear wave elastography. Insights Imaging. (2020) 11:28. doi: 10.1186/s13244-020-0841-4 32128718 PMC7054526

[19] Zhang S Lu C Zhao Q Xiang Y He W Qu Y . The association between a history of induced abortion for nonmedical reasons and maternal and neonatal perinatal outcomes: a retrospective cohort study. J Matern Fetal Neonatal Med. (2025) 38:2466207. doi: 10.1080/14767058.2025.2466207 39988368

[20] Pang Q Peng L Wu J Wang Y Zhang R Liu Z . Comparison of interpretable machine learning models using systemic inflammation index to predict preterm birth in gestational diabetes mellitus. Int J Womens Health. (2026) 18:541610. doi: 10.2147/IJWH.S541610 41710144 PMC12912001

[21] Ugwudike B Kwok M. Update on gestational diabetes and adverse pregnancy outcomes. Curr Opin Obstet Gynecol. (2023) 35:453–9. doi: 10.1097/GCO.0000000000000901 37560815

[22] Ende HB Lozada MJ Chestnut DH Osmundson SS Walden RL Shotwell MS . Risk factors for atonic postpartum hemorrhage: a systematic review and meta-analysis. Obstet Gynecol. (2021) 137:305–23. doi: 10.1097/AOG.0000000000004228 33417319 PMC8336570

[23] Bellamy L Casas JP Hingorani AD Williams D. Type 2 diabetes mellitus after gestational diabetes: a systematic review and meta-analysis. Lancet. (2009) 373:1773–9. doi: 10.1016/S0140-6736(09)60731-5 19465232

[24] Allen LH. Biological mechanisms that might underlie iron's effects on fetal growth and preterm birth. J Nutr. (2001) 131:581S−9S. doi: 10.1093/jn/131.2.581S 11160591

[25] Omotayo MO Abioye AI Kuyebi M Eke AC. Prenatal anemia and postpartum hemorrhage risk: a systematic review and meta-analysis. J Obstet Gynaecol Res. (2021) 47:2565–76. doi: 10.1111/jog.14834 34002432 PMC9258034

[26] WOMAN-2 trial collaborators. Maternal anaemia and the risk of postpartum haemorrhage: a cohort analysis of data from the WOMAN-2 trial. Lancet Glob Health. (2023) 11:e1249–9. doi: 10.1016/S2214-109X(23)00245-0 37390833 PMC10353972

[27] Guasch E Gilsanz F. Massive obstetric hemorrhage: current approach to management. Med Intensiva. (2016) 40:298–310. doi: 10.1016/j.medine.2016.02.003 27184441

